# Effects of direct-fed microbials supplementation on in vitro and ex vivo ruminal fermentation and nutrient degradability in lactating Holstein dairy cows

**DOI:** 10.1093/tas/txae162

**Published:** 2024-12-14

**Authors:** Adeoye O Oyebade, Kathy Arriola, Oscar Queiroz, Bruno I Cappellozza, Diwakar Vyas

**Affiliations:** Department of Animal Sciences, Institute of Food and Agricultural Sciences, University of Florida, Gainesville, FL 32611, USA; Department of Animal Sciences, Institute of Food and Agricultural Sciences, University of Florida, Gainesville, FL 32611, USA; Novonesis, Lyngby 2800, Denmark; Novonesis, Lyngby 2800, Denmark; Department of Animal Sciences, Institute of Food and Agricultural Sciences, University of Florida, Gainesville, FL 32611, USA

**Keywords:** *Bacilli*, direct-fed microbial, *Lactobacilli*, neutral detergent fiber, *Propionibacterium*

## Abstract

We conducted two experiments to evaluate the effect of direct-fed microbials (**DFM**) on fermentation parameters and nutrient degradability with two different approaches using rumen fluid from lactating Holstein dairy cows. In Exp. 1, three doses of a DFM containing *Lactobacillus animalis* and *Propionibacterium freudenreichii* (**PRO-A**) at doses of 3.9 × 10^6^, 7.8 × 10^6^, and 11.7 × 10^6^ CFU or a DFM containing PRO-A*, Bacillus subtilis*, and *B. licheniformis* (**PRO-B**) at doses of 15.2 × 10^6^, 30.4 × 10^6^, and 45.6 × 10^6^ CFU were incubated using corn silage as substrate and pooled rumen fluid from three-rumen fistulated lactating Holstein cows. Dry matter and NDF degradability, gas production, and rumen pH were measured over a 24-h period. In Exp. 2, three ruminally cannulated multiparous cows (165 ± 63 DIM) were used in a 3 × 3 Latin square design. Each experimental period was of 28 d. All cows received a corn silage-based TMR (basal diet), and were assigned to: 1) Control (**CON**), 2) PRO-A: Basal diet top-dressed with PRO-A at 3 × 10^9^ CFU/day, and 3) PRO-B: Basal diet top-dressed with PRO-B at 11.8 × 10^9^ CFU/day. An ex vivo study (Exp. 2) was conducted using rumen fluid collected during wk 4 of each experimental period from experimental animals. Treatments included: CON, PRO-A, PRO-B, each of which utilized rumen fluid from donor cows given respective treatments. Another set of rumen fluid from PRO-A and PRO-B cows were dosed with additional dose of respective DFM, resulting in two more treatments (PRO-A+ and PRO-B+). In Exp. 1, linear effects (*P* = 0.03) were observed on in vitro NDF degradability following DFM incubation. In Exp. 2, no treatment effects were observed on DM and NDF digestibility. In summary, DFM increased DM and NDF degradability in vitro using rumen fluid from cows not exposed to DFM; however, no effects were observed under ex vivo experimental conditions when rumen fluid was collected from cows consuming DFM.

## Teaser

Our research investigates in vitro efficacy of direct-fed microbials (**DFM** ) on rumen fermentation and nutrient digestibility in dairy cows, revealing that while DFM enhances fiber breakdown in laboratory settings, these benefits were not observed in cows already consuming DFM in their diets.

## INTRODUCTION

Bacterial direct-fed microbials (**DFM**) supplementation has been extensively investigated in both dairy (Oyebade et al., 2023a, b) and beef cattle diets (Krehbiel et al., 2003). Recently, the focus has shifted towards dietary supplementation of multispecies bacterial DFM, as synergy among the different genus, species, and strains might lead to additive benefits to the host when compared with single species and/or strain DFM supplementation. Bacterial species explored as constituents of DFM for ruminants include lactic acid-producing bacteria (*Lactobacillus* spp. and *Enterococcus* spp.), lactic acid-utilizing bacteria (*Propionibacterium* spp.), and *Bacillus* spp. (Seo et al., 2010; McAllister et al., 2011). Among lactate producers, *Lactobacillus spp.* is most commonly used to sustain rumen production of lactic acid, a substrate which is expected to support the proliferation of lactate utilizers, like *Propionibacterium* spp., which converts lactate to propionate, the main precursor for gluconeogenesis in ruminants (Huntington, 1990).

Studies that supplemented multispecies combination of *Lactobacillus spp.* and *Propionibacterium spp*. to lactating dairy cows (Boyd et al., 2011; West and Bernard, 2011; Dickey, 2016) at doses ranging between 3 × 10^9^ and 4 × 10^9^ CFU/d and reported increased yields of milk, milk fat, and/or milk protein, showing potential benefits to supplementing multispecies DFM. However, the results are inconsistent as other studies (Raeth-Knight et al., 2007; Ferraretto and Shaver, 2015; Lawrence et al., 2021) using similar combination of DFM species at a dose of 3 × 10^9^ CFU/d reported no effect on milk composition and yield. We can speculate that the inclusion of *Bacillus* spp. might introduce a synergy that could improve the consistency of these beneficial effects in multispecies DFM such as *Lactobacillus* spp. and *Propionibacterium* spp. Bacilli are spore-forming bacteria that survive through the most challenging conditions during feed preparation (Cappellozza et al., 2023b) and have ability to produce different types of enzymes (Schallmey et al., 2004) which also makes them suitable candidates as feed additive for lactating dairy cattle diets (Qiao et al., 2010; Sun et al., 2013).

However, to our knowledge, few studies have evaluated the response of different DFM in a dose–response manner using different in vitro experimental models. These in vitro studies differ in the strains and species of DFM used, the dosage rates applied, the substrates utilized, and the duration of the incubation (Cappellozza et al., 2023a; Silva et al., 2024), making it difficult to directly compare their results. For example, some studies used *Lactobacillus*-based or *Bacillus*-based DFM (Jeyanathan et al., 2016; Cappellozza et al., 2023a), while others used DFM at varying doses, using forages, cereal grains or TMR as substrates (Pan et al., 2022; Dhakal et al., 2023). Thus, the response of rumen fermentation to different DFM supplements and dosages remains unclear and inconsistent.

The in vitro rumen fermentation has proven to be an economical model which can be used to quickly access the potential of rumen modifiers. While the model is not perfect, it provides insights that can be used to predict what could be obtainable in live animal trials. In vitro models, however, often use short incubation periods (e.g., 24 h), which may not allow DFM to exert its full effects, such as modifying the rumen microbiome and reflecting the benefits of this modification. In contrast, ex vivo models—where animals are fed DFM for a longer duration (typically over 21 d) before rumen fluid collection—might provide a more comprehensive view of microbial and fermentation changes.

Therefore, the objective of this study was to evaluate the effect of bacterial DFM doses on in vitro fermentation characteristics using rumen fluid from lactating dairy cows; 1) not consuming DFM (Exp. 1) and 2) fed diets supplemented with DFM (ex vivo model; Exp. 2). We hypothesized that DFM would improve rumen fermentation efficiency, resulting in increased propionate production, lower A:P ratio, and lower methane production per unit of feed degraded regardless of the experimental approach used.

## MATERIALS AND METHODS

All animal care and experimental procedures for this study were approved by the University of Florida Institutional Animal Care and Use Committee (Protocol number: 201810520).

### Experiment 1: In Vitro Dose-Response

The experimental treatments evaluated herein were control (**CON**; no probiotic inoculation), inoculation of a DFM containing *Lactobacillus animalis* 506 and *Propionibacterium freudenreichii* 507 (**PRO-A**), or inoculation of a DFM containing *L. animalis*, *P. freudenreichii*, *Bacillus licheniformis* 809, and *B. subtilis* 810 (**PRO-B**). Both PRO-A and PRO-B were tested under three different dosages, representing 3.9 × 10^6^, 7.8 × 10^6^, and 11.7 × 10^6^ CFU of PRO-A (1.625, 3.250, and 4.875 mg/0.5 g of substrate, respectively) and 15.2 × 10^6^, 30.4 × 10^6^, and 45.6 × 10^6^ CFU of PRO-B (1.625, 3.250, and 4.875 mg/0.5 g of substrate, respectively). The calculation of the DFM dose to be incubated into each jar was based on a rumen capacity of 80 L and the recommended standard dose of each DFM on a daily basis (total dose of 3.0 × 10^9^ and 11.8 × 10^9^ CFU/head per day for PRO-A and PRO-B, respectively), according to the manufacturer recommendation. Dried ground corn silage was used as substrate for these assays (Table 1).

**Table 1.** Nutrient composition of the corn silage used as substrate (Experiment 1)

Nutrient	Percentage, % (DM basis)
Dry matter, % fresh	33.2
Net energy, Mcal/kg	1.63
Crude protein	7.19
aNDF	36.5
ADF	21.8
Lignin	4.0
Starch	37.23
Ash	4.01

#### Donor animals and inoculum collection

 Three-rumen fistulated multiparous lactating Holstein cows (DIM 120 ± 50; BW 680 ± 75 kg) were used as the inoculum source for the present study. The donor cows were fed ad libitum twice daily (0600 and 1400 h) a corn silage-based TMR (Table 2). Between 2 and 3 h after morning feeding, rumen fluid was manually collected, filtered through a two-layer cheesecloth, and pooled into prewarmed thermos flasks kept at 39 °C. No additives, such as prebiotics, probiotics, enzymes, ionophores, and/or nonionophores (virginiamycin, for example) were added in the TMR offered to donor dairy cows. Donor cows were on this TMR diet for 60 d prior to first rumen fluid collection. Thermos flasks containing pooled rumen fluid were kept airtight until transported to the laboratory for final filtration into two more layers of cheesecloth (total of four layers).

**Table 2.** Ingredient and chemical composition of the diet fed to rumen fluid donors (Experiment 1)

Item	Inclusion, %
*Ingredients, % of diet DM*	
Corn silage	38.2
Wheat straw	4.0
Ground corn	27.3
Soybean meal, 44%	14.5
Citrus pulp	9.2
Mineral and Vitamin mixture^1^	5.0
Palmit-80^2^	1.8
*Dietary DM, %*	*54.4*
*Nutrient composition (DM basis)*	
Net energy^3^, Mcal/kg	1.83
OM, %	93.0
Crude protein, %	16.5
aNDF, %	25.1
ADF, %	15.3
Starch, %	30.4
Ash, %	7.0

^1^Vitamin mineral mixture (DM basis): Ca, 7.44%; P, 1.60%; Mg, 2.52%; K, 0.21%, S, 0.44%; Na, 8.13%; Cl, 3.30%; Biotin, 2.17 ppm; Fe, 1,221.5 ppm; Zn, 1,450.35 ppm; Cu, 220.32 ppm; Mn, 1,180.14 ppm; Se, 7.33 ppm; Co, 22.51 ppm; I, 12.43 ppm; Vitamin A, 273.22 KIU/kg; Vitamin D, 63.95 KIU/kg; Vitamin E, 546.44 IU/kg.

^2^Global Agri Trade Corporation, Long Beach, CA.

#### In vitro batch culture experiment

The corn silage (1 mm, 0.50 g) was dried in the lab at 105 °C for 24 h and then weighed into Ankom F-57 filter bags (Ankom Technology, Macedon, NY). Before sample weighing, empty F-57 filter bags were prerinsed in acetone for 5 min and then air dried per manufacturer instructions. Bags were sealed with an Uline Tabletop Poly Bag Sealer (Impulse type AIE-200, American International Electronics, Kenneth City, FL) and immediately placed into 160 mL serum vials. Four replicate substrate and inoculum were incubated in separate vials per treatment per run.

The inoculum collected from each cow was pooled and added to a buffered prewarmed (39 °C) media (Goering and Van Soest, 1970) in a 1:2 ratio of rumen fluid:artificial saliva (McDougall, 1948). The media was continuously infused with carbon dioxide (**CO**_**2**_) to maintain an anaerobic environment for the rumen fluid inoculum. Buffered rumen fluid (52 mL) was added to the 160-mL serum vials containing Ankom bags and a continuous stream of CO_2_ was flushed into the vials during the whole inoculation process. Vials were closed with rubber stoppers and crimped with aluminum seals. Vials were immediately placed in an air-forced incubator at 39 C with a shaking system for 24 h for the study.

#### In vitro dry matter (IVDMD) and neutral detergent fiber (IVNDFD) digestibility

A total of four substrate-containing Ankom bags were incubated per treatment per run. The blank correction was used to estimate net gas production as well as for IVDMD and IVNDFD. Incubations were terminated by placing the bottles on ice after 24 h of incubation, with bags being taken out of vials, washed with tap water, and then dried in a forced-air oven at 60 °C for 48 h. Dried residues were weighed and the remaining amount was used to estimate apparent IVDMD. Bags were then analyzed for aNDF using sodium sulfite and heat-stable α-amylase in an Ankom-200 fiber analyzer (Ankom Technology Corp.). Then, bags were dried once again at 60 °C for 48 h and weighed to calculate IVNDFD.

#### In vitro gas production

Four serum flasks were independently incubated for each of 24 and 48 h incubations, and headspace gas pressure was measured after the incubation periods (at 24 and 48 h only). Gas pressure was corrected for gas pressure in blank flasks and converted to gas volume based on the current lab conditions using the following equation:


$$\begin{array}{l} Gas\;volume\;\left(mL\right)=\left(Gas\;pressure\;\left(\;psi\right)\times 4.8843\right)+3.1296;\;\;\;r^{2}\;\;\;=\;\;\;0.97 \end{array}$$

### Experiment 2: Ex Vivo

#### Experimental animals and treatments

Three-rumen-cannulated lactating multiparous Holstein cows with an average (mean ± SD) 165 ± 63 DIM, 25 ± 9 kg milk/d, 880 ± 54 kg BW were used in a 3 × 3 Latin square design study. Cows were housed together in a sand-bedded free stall barn with each cow assigned to separate individual feeding gates (Calan Broadbent feeding system, American Calan Inc., Northwood, NH) and trained to eat from the assigned bunk. The experimental pens were equipped with 2 rows of fans (1 fan/6 linear meters) and low-pressure nozzles were mounted over the feeding bunk, facing away from the feed and onto the cows to help with cooling. Fans and nozzles were activated whenever ambient temperature reached 21 °C. Beddings was cleaned twice a day, and manure was flushed from pen twice daily via automated flushing system.

The treatments were: 1) Control diet with no DFM supplement (CON), 2) CON with the addition of PRO-A (*L. animalis* and *P. freudenreichii*) supplemented at 3 × 10^9^ CFU/head per day (PRO-A), and 3) CON with the addition of PRO-B (*L. animalis*, *P. freudenreichii*, *B. licheniformis*, and *B. subtilis*) supplemented at 11.8 × 10^9^ CFU/head per day (PRO-B). Lactose powder was used as carrier for both DFM supplements. The DFM supplements were mixed with dried molasses before top-dressed on the TMR offered in the morning, while the CON group received only molasses as top-dress. Each treatment period lasted 28 d with the last 14 d used for data and sample collection. (Materials and Methods and Results from in vivo *study* presented in Supplementary Material S1). All cows received a corn silage-based TMR (Table 3), which was formulated using NRC (2001). Diets were fed twice daily at 0600 and 1200 h, offering 60% and 40% of the total feed offered daily at respective times. Feed was offered to ensure ad libitum intake with a minimum daily refusal of 5%. Water was available ad libitum throughout the experimental period.

**Table 3.** Ingredient and chemical composition of the basal diet used in feeding trial (Experiment 2)

Item	Inclusion, %
*Ingredients, % of diet DM*	
Corn silage	47.1
Corn grain, ground shelled	18.8
Soybean meal, 44%	15.1
Citrus pulp	4.7
Whole cottonseed	7.4
Palmit-80^1^	1.7
Mineral and vitamin mix^2^	5.2
*Dietary DM, %*	*47.1*
*Nutrient composition (DM basis)*	
Net energy,^3^ Mcal/kg	1.85
OM, %	92.5
Crude protein, %	17.7
aNDF, %	34.2
Forage aNDF, %	20.8
ADF, %	17.3
Starch, %	27.8
NFC, %	33.3
Crude fat, %	7.3
Ash, %	7.5
Macro-minerals	
Calcium, %	0.65
Phosphorous, %	0.41
Magnesium, %	0.30
Potassium, %	1.06
Sulphur, %	0.19
Sodium, %	0.44
Chloride, %	0.35

^1^Global Agri Trade Corporation, Long Beach, CA.

^2^Vitamin mineral mixture (DM basis): Ca, 7.44%; P, 1.60%; Mg, 2.52%; K, 0.21%, S, 0.44%; Na, 8.13%; Cl, 3.30%; Biotin, 2.17 ppm; Fe, 1,221.5 ppm; Zn, 1,450.35 ppm; Cu, 220.32 ppm; Mn, 1,180.14 ppm; Se, 7.33 ppm; Co, 22.51 ppm; I, 12.43 ppm; Vitamin A, 273.22 KIU/kg; Vitamin D, 63.95 KIU/kg; Vitamin E, 546.44 IU/kg.

^3^Calculated using chemical analysis of dietary ingredients according to NRC (2001).

#### Ex vivo batch culture assay

Rumen fluid was collected 2 to 3 h after morning feeding from rumen-cannulated lactating dairy cows used in the 3 × 3 Latin square design during the last week of each experimental period (days 22, 24, 26, and 28). The experiment was repeated in three independent periods, all of which corresponded to the 3 periods of the feeding trial. Each period of this in vitro study had 4 independent runs. The corn silage-based diet fed to cows was used as substrate for in vitro batch culture assy. Since rumen fluid was collected from cannulated cows in Experiment 2, treatments in this experiment corresponded to the dietary treatments cannulated cows were receiving; CON, PRO-A (3 × 10^9^ CFU/head per day), and PRO-B (11.8 × 10^9^ CFU/head per day). Additional treatments included in Exp. 2 were PRO-A+ and PRO-B+, comprising an additional dose of 3.9 × 10^6^ CFU and 15.2 × 10^6^ CFU of PRO-A and PRO-B, respectively. This approach was performed to minimize any risk that the bacteria contained in the DFM supplements would not get established within the rumen of supplemented cows.

For every incubation, 0.50 g of ground TMR samples was weighed into dried F-57 Ankom bags (Ankom, Macedon), sealed, and placed into 160-mL glass vials. Ground TMR (1-mm) was used as substrate, except for starch digestibility estimation, where TMR was ground to 4-mm following the procedures of Ferraretto et al. (2015). All treatments were incubated in triplicates for each independent run, except for starch digestibility assessment, where treatments were incubated in quadruplicates for each independent run. Blank vials with bags containing no substrates were incubated in duplicates for all treatments to correct for changes in the weight of incubated bags.

Rumen fluid was collected from donor cows, filtered through 4 layers of cheesecloth, and mixed with prewarmed (39 °C) buffer (artificial saliva) at a ratio of 1:2 rumen fluid to buffer. This media was continuously stirred and infused with CO_2_ to maintain an anaerobic condition. The additional treatments, PRO-A+ and PRO-B+, were inoculated just prior to sealing and immediate incubation. Buffered ruminal fluid (52 mL) was dispensed into each vial, immediately covered with rubber stoppers, and sealed with aluminum crimp caps. Samples were incubated in forced-air incubator at 39 °C for 24 h. For starch digestibility estimation, samples were incubated in forced-air incubator at 39 °C for 7 h. The 7-h incubation time-point is the industry standard for measuring starch disappearance as described by Saylor et al. (2020). For all analyses, fermentation was terminated by placing vials on ice.

### Sampling and Analysis

#### Degradability, gas volume, and methane concentration

After 24 h of incubation, bags were rinsed and dried at 60 °C for 48 h for estimation of DM degradability. Dried residues were analyzed for NDF (Van Soest et al., 1991) using an Ankom 200 Fiber Analyzer (Ankom, Macedon, NY). For starch analysis, composites were made from the 7-h incubation residues by pooling two replicates from the quadruplicates of each sample. Composites were analyzed for starch concentration, as described by Hall (2015). Gas production was measured at 0, 3, 6, 9, 12, and 24 h postincubation using a pressure transducer (Mauricio et al., 1999) and the following equation was derived under our lab condition to convert gas pressure to volume:


$$\begin{array}{l} Gas\;volume\;\left(mL\right)=\left(Gas\;pressure\;\left(\;psi\right)\times 4.8843\right)+3.1296 \end{array}$$

Gas production was corrected using values taken from blank vials. After the 24 h incubation, 20 mL gas samples were collected from each vial and stored in sealed vacuumed glass vials for methane analysis. Methane concentrations were estimated by gas chromatography (Agilent 7820A GC, Agilent Technologies, Palo Alto, CA) using a flame ionization detector and a capillary column (Plot Fused Silica 25 m × 0.32 mm, Coating Molsieve 5A, Varian CP7536), as described by Pech-Cervantes et al. (2019).

Ammonia, pH, and VFA analysis. Residual inoculum from each 24 h incubation vial was analyzed for pH. Samples were acidified using 50% H_2_SO_4_ (1% vol/vol) and centrifuged at 8,000 × *g* for 15 min at 4 °C. Supernatant was frozen at −20 °C until analyzed for VFA concentration using an HPLC (Hitachi, L2400, Tokyo, Japan) fitted with a Bio-Rad Aminex HPX-87H column (Bio-Rad Laboratories, Hercules, CA), as described by others (Romero et al., 2015). Unacidified residual inoculum was analyzed for concentration of NH_3_-N as previously described by Monteiro et al. (2019).

### Statistical Analysis

Data from Exp. 1 was analyzed with the GLIMMIX procedure of SAS (version 9.4; SAS Institute Inc., Cary, NC) according to the following model:


$$\begin{array}{l} Y_{ij}=\;\mu +D_{i}+R_{j}+E_{ijk} \end{array}$$

where *µ* = overall mean; $D_{i}$ = fixed effect of dose, *i* = 1 to 4; $R_{j}$ = random effect of run, *j* = 1 to 3; $E_{ijkl}$ = residual random error of experiment. For each DFM product, analysis was performed separately as the goal was to find the best dose-response for Exp. 2. Within each DFM, an analysis of the effects of probiotics (dose 0 vs average of other doses), as well as linear and quadratic effects of doses was also performed.

Data from Exp. 2 were analyzed using the GLIMMIX procedure of SAS (version 9.4; SAS Institute Inc.), according to the following model:


$$\begin{array}{l} Y_{ijk}=\;\mu +T_{i}+P_{j}+C{\left(R\right)}_{kl}+{\left(T\times P\right)}_{ij}+E_{ijkl} \end{array}$$

where *µ* = overall mean; $T_{i}204T_{i}$ = fixed effect of treatment, *i* = 1 to 5; $P_{j}$ = fixed effect of period, *j* = 1 to 3; $C{\left(R\right)}_{kl}$ = random effect of Cow *k* (*k* = 1 to 3) nested in run (*l* = 1 to 4); ${\left(T\times P\right)}_{ij}$ = treatment × period interaction; $E_{ijkl}$ = residual random error of experiment. Gas production was analyzed as repeated measurements, where treatments, period, and sampling hours were used as fixed factors, whereas cow nested in run was included as the random factor. An autoregressive variance–covariance structure was used to account for the correlations among repeated measures. Normality of the residuals was assessed using the Shapiro–Wilk test while the Breusch–Pagan test was used to evaluate heteroscedasticity in the residuals. Log transformation was applied when necessary to meet the assumptions of normality and homoscedasticity. Sampling hour was used as a repeated measure. Treatment effects were declared significant at *P* ≤ 0.05, and tendencies were declared at 0.05 < *P* ≤ 0.10.

## RESULTS

### Experiment 1

No differences were observed on in vitro DM degradability, gas production, and pH when corn silage was used as substrate (*P* ≥ 0.20; Table 4). However, NDF degradability was linearly increased (*P* = 0.03) in response to incremental doses of PRO-A, and the quadratic effect tended to be significant (*P* = 0.09). Similarly, NDF degradability was linearly increased with incremental inclusion of PRO-B (*P* = 0.03) while no treatment effects were observed on pH, DM degradability, and total gas production (*P* > 0.10; Table 4). Based on the results observed in this experiment, we selected lowest dose to be used for animal feeding and experiment 2.

**Table 4.** Effects of incubation with different doses of DFM on in vitro fermentation traits (Experiment 1)^1^

Item	Doses, mg				SEM	*P =* ^2^		
	0	1.625	3.250	4.875		DFM	L	Q
*PRO-A*								
pH	6.25	6.23	6.17	6.23	0.045	0.44	0.57	0.36
DM degradability, %	41.7	42.6	41.0	40.0	1.15	0.72	0.20	0.40
NDF degradability, %	25.8	31.4	30.4	31.1	1.43	<0.01	0.03	0.09
Total gas production, mL/g dry matter	129.7	134.9	135.5	134.0	4.52	0.33	0.50	0.46
*PRO-B*								
pH	6.25	6.16	6.24	6.15	0.040	0.16	0.24	0.93
DM degradability, %	41.7	42.8	42.0	43.2	0.87	0.39	0.37	0.92
NDF degradability, %	25.8	29.4	28.3	31.6	1.53	0.04	0.03	0.92
Total gas production, mL/g dry matter	129.7	137.7	133.1	134.1	5.05	0.37	0.70	0.49

^1^Treatments for PRO-A included 3.9, 7.8, and 11.7 × 10^6^ CFU of PRO-A (1.625, 3.250, and 4.875 mg, respectively). Treatments for PRO-B included 15.2, 30.4, and 45.6 × 10^6^ CFU of PRO-B (1.625, 3.250, and 4.875 mg, respectively).

^2^Contrast analysis: DFM = 0 vs. 1.625 + 3.250 + 4.875; L = linear effect of dose; Q = quadratic effect of dose.

### Experiment 2

#### Ex vivo digestibility, gas production, rumen fermentation traits, and methane

Treatments had no effect (*P *> 0.10) on ex vivo DM, NDF, and starch degradability (Table 5). Similarly, treatments had no effect (*P *> 0.10) on gas volume, gas volume per unit of DM degraded and, methane concentration. Treatments likewise had no effect on ruminal NH_3_-N concentration, pH, total VFA concentration and proportion of individual VFAs, except for butyrate (Table 6). Both PRO-A and PRO-B supplements decreased (*P *≤ 0.05) the proportion of butyrate vs. CON (Table 6).

**Table 5.** Nutrient digestibility and gas production in response to DFM treatments in Experiment 2

Parameter	Treatment^1^					SEM	Contrast *P*-value^2^		
	CON	PRO-A	PRO-A+	PRO-B	PRO-B+		C vs. A	C vs. B	A vs. B
*Digestibility, %*									
DM	52.6	53.3	52.9	53.1	52.4	0.90	0.81	0.97	0.73
NDF	41.0	41.8	41.6	41.1	42.0	0.86	0.69	0.78	0.89
Starch	58.9	57.6	59.5	62.0	56.6	4.21	1.00	1.00	0.90
*Gas Parameters*									
Gas volume, mL/g DM	85.7	78.9	76.8	83.1	79.6	4.10	0.20	0.55	0.50
Gas volume, mL/g DMD	162.8	149.6	143.8	155.3	151.9	7.73	0.14	0.44	0.46
Methane, mg/g DM	6.1	5.7	4.9	5.8	5.5	0.58	0.42	0.73	0.54
Methane, mg/g DMD	11.6	10.7	9.3	10.8	10.5	1.04	0.33	0.67	0.57

^1^All treatments used the same corn silage-based TMR (basal diet) in in vitro batch culture, using either: rumen fluid from dairy cow fed basal diet without DFM (CON), rumen fluid from dairy cow fed basal diet top-dressed with 3 × 10^9^ CFU/cow per day of a DFM containing *Lactobacillus animalis* and *Propionibacterium freudenreichii* (PRO-A), PRO-A rumen fluid with additional dose (1.62 mg/mL) of PRO-A (PRO-A+), rumen fluid from dairy cow fed basal diet top-dressed with 11.8 × 10^9^ CFU/cow per day of a DFM containing *L. animalis*, *P. freudenreichii, Bacillus subtilis,* and *B. licheniformis* (PRO-B), PRO-B rumen fluid with additional dose (1.62 mg/mL) of PRO-B (PRO-B+).

^2^C vs. A = CON vs. PRO-A and PRO-A+, C vs. B = CON vs. PRO-B and PRO-B+, A vs. B = PRO-A and PRO-A + vs. PRO-B and PRO-B+.

**Table 6.** Ammonia-N, pH, and volatile fatty acid production in response to DFM treatments in Experiment 2

Parameter	Treatment^1^						Contrast *P*-value^2^		
	CON	PRO-A	PRO-A+	PRO-B	PRO-B+	SEM	C vs. A	C vs. B	A vs. B
Ammonia-N, mg/dL	48.5	46.6	45.2	5.6	46.8	2.18	0.55	1.00	0.33
Rumen pH	6.4	6.4	6.4	6.4	6.4	0.05	0.77	0.96	0.70
Total VFA, mmol	105.5	115.2	112.4	104.6	102.2	7.50	0.37	0.99	0.18
Individual VFA, mol/100 mol									
Acetate	51.1	51.0	51.4	5.3	5.9	0.37	0.99	0.30	0.19
Propionate	23.0	23.6	23.6	23.7	23.5	0.53	0.55	0.50	0.92
Butyrate	14.3	13.8	13.6	13.8	13.7	0.21	0.05	0.04	0.92
Isobutyrate	2.8	2.7	2.7	2.9	2.8	0.13	0.86	0.93	0.39
Valerate	4.1	4.1	4.1	4.3	4.2	0.11	0.99	0.39	0.11
Isovalerate	4.7	4.7	4.7	5.1	5.0	0.17	0.98	0.17	0.11
Acetate:Propionate	2.2	2.2	2.2	2.2	2.2	0.06	0.60	0.57	0.94

^1^All treatments used the same corn silage-based TMR (basal diet) in in vitro batch culture, using either: rumen fluid from dairy cow fed basal diet without DFM (CON), rumen fluid from dairy cow fed basal diet top-dressed with 3 × 10^9^ CFU/cow per day of a DFM containing *Lactobacillus animalis* and *Propionibacterium freudenreichii* (PRO-A), PRO-A rumen fluid with additional dose (1.62 mg/mL) of PRO-A (PRO-A+), rumen fluid from dairy cow fed basal diet top-dressed with 11.8 × 10^9^ CFU/cow per day of a DFM containing *L. animalis*, *P. freudenreichii, Bacillus subtilis,* and *B. licheniformis* (PRO-B), PRO-B rumen fluid with additional dose (1.62 mg/mL) of PRO-B (PRO-B+).

^2^C vs. A = CON vs. PRO-A and PRO-A+, C vs. B = CON vs. PRO-B and PRO-B+, A vs. B = PRO-A and PRO-A + vs. PRO-B and PRO-B+.

## DISCUSSION

To our knowledge, few studies have evaluated the effects of multispecies bacterial-based DFM on in vitro fermentation traits and nutrient degradability using different DFM doses. Boyd et al. (2011) observed greater digestibility of CP and NDF when a corn silage, alfalfa hay and rye silage-based TMR diet (having 48.1% forage, 37.8% NDF, and 17.9% CP) was supplemented with 4 × 10^9^ CFU/h/d of *L. acidophilus* and *P. freudenreichii*, which translated to increased milk yield and milk protein yield. West and Bernard (2011) fed corn silage and alfalfa hay-based TMR diet (having 39.1% forage, 26.7% NDF, and 20.3% CP) and supplemented 2 × 10^9^ CFU/hd/d of *P. freudenreichii* NP24 alongside either *L. acidophilus* NP51 or *L. acidophilus* NP51 and NP45. The authors reported an average effect of the DFM supplements as tending to increase milk yield, with a significant increase in milk fat yield, FCM yield and ECM yield. Recently, Maderal et al. (2022) reported quadratic responses on in vitro fermentation traits when a combination of *B. licheniformis* and *B. subtilis* were incubated with a sorghum silage-based substrate at a 1, 5, and 10 times the recommended dose (8 × 10^4^ CFU/mL), with the highest total VFA, NH_3_-N and total gas production observed when 5-times the recommended dosage was incubated. Pech-Cervantes et al. (2019) observed greater in vitro DM and NDF degradability in corn silage and bermudagrass silage in presence of expansin-like proteins from *B. subtilis*. In agreement with previous findings, we have demonstrated linear effects on in vitro NDF degradability with both DFMs. The effects on increasing fiber degradability following PRO-B incubation may be attributed to the presence of cellulases from *B. licheniformis* and expansin-like proteins from *B. subtilis*; however, cellulase activity was not measured in the current study. Lastly, no further differences were reported in the other parameters evaluated in vitro, also supporting the previous study with *Bacillus* spp. (Maderal et al., 2022).

The ex vivo study presented here (Exp. 2) was conducted using rumen fluid collected from individual cows supplemented with each treatment in a 3 × 3 Latin square design. When compared with the regular pooling of rumen fluids from different cows for in vitro assays, ex vivo approach brings an opportunity to account for the direct in vivo response of each individual cow fed the specific DFM overtime, and the use of a Latin square design helps to ensure that these individual in vivo responses of each cow is accounted for all treatments, thereby reducing the variability within the study on rumen fermentation parameters including gas production, VFA, and nutrient degradability. However, contrary to our expectations, no effects of DFM were observed on digestibility of DM, NDF, and starch using ex vivo approach. The results observed in this study, while not in agreement with our findings in Experiment 1 but are in agreement with previous studies (Meale et al., 2014; Dhakal et al., 2023). Chen et al. (2020) observed greater DMD with *P. freudenreichii* T31 (treated with 450 µL DFM culture having concentration of 2 × 10^8^ to 4 × 10^8^ CFU/100 µL) using high forage substrate (60% grass silage, 16% barley, 16% wheat, and 8% soybean meal) under an in vitro system. To our knowledge, few studies have evaluated the combinations presented herein on in vitro and ex vivo settings using different substrates. The utilization of *Bacillus* spp. as a DFM has been gaining attention and interest of different players in the production chain, mainly due to their enzyme-producing ability that has led to greater in vitro DM and NDF degradability, as well as total gas production when forage-based and commercial dairy TMR were used as substrates (Pan et al., 2022; Cappellozza et al., 2023a). One may speculate that the differences in results between Exp. 1 and 2 could be associated with the different substrates used, being corn silage only for Exp. 1 and a complete dairy cow TMR in Exp.2. The corn silage in Exp. 1 has a greater percentage of aNDF (44.1%) than the Exp. 2 TMR (34.2%). The lower NDF in the TMR might have reduced the opportunity available for improvement, thus resulting in a lower margin of improvement. In addition, the nutrient composition of Exp 2. substrate (a TMR) was more balanced, which would allow rumen microbes in all treatment groups to operate near maximum efficiency, which could reduce the opportunity for additional improvement by supplemented DFM. Another plausible explanation is the differences in the diet fed to the donor cows, where the donor diet in experiment 1 contains lesser forage and with lower NDF and higher starch than the donor diet in experiment 2. The authors, Cappellozza et al. (2023a), in an in vitro experiment supplemented TMR substrates from seven different dairies, having NDF concentrations varying from 25.1 to 34.1 %DM, either with or without *B. licheniformis* and *B. subtilis* at a dose of ~7.13 × 10^6^, and reported a significant increase in average NDFD for the DFM group relative to the untreated control. The highest NDF concentration from the TMR substrates used in this study were close to those of the TMR substrate used in Exp. 2 of the current study, implying that diet composition alone does not fully explain the inconsistency observed between Exp. 1 and 2 of the current study Therefore, additional studies are warranted to understand not only the potential benefits of adding different DFM under a wider range of substrates having varying nutrient composition but also to evaluate the DFMs under in vitro, ex vivo or in vivo while making comparisons easier through keeping factors such as substrate similar across models, which is a limitation in the current study.

Supplemental bacterial DFM had no effect on rumen pH, individual proportion and total VFA, except for molar proportion of butyrate. Greater molar proportion of propionate was reasonably expected when propionate-producing DFM is supplemented, but results have been variable between in vitro (Alazzeh et al., 2012; Meale et al., 2014; Chen et al., 2020; Dhakal et al., 2023) and in vivo settings (Vyas et al., 2014b; Philippeau et al., 2017; Lawrence et al., 2021), such that effects observed on VFA parameters in vitro sometimes differ from those observed in vivo. The propionate formation pathways reduce pyruvate to propionate, making them competitive pathways for H_2_ in the rumen (Ungerfeld, 2020). Given that H_2_ is an indispensable precursor in the formation of CH_4_, an increase in propionate concentration implies a competitive reduction in CH_4_ production (Moss et al., 2000; Jeyanathan et al., 2014). As such, propionate-producing bacteria such as *Propionibacterium spp.* have been researched and identified as potential supplements to mitigate CH_4_ production within the rumen (Vyas et al., 2014a, b; Vyas et al., 2015). The supplemental DFM fed in the current study however had no effect on gas volume and CH_4_ production, which is in agreement with previous studies that supplemented *P. freudenreichii* DFM in in vitro culture systems and reported no change in gas production (Meale et al., 2014) and CH_4_ concentration (Chen et al., 2020). Compared to the results of the current study; Meale et al. (2014) and Alazzeh et al. (2012) reported a decrease in CH_4_ production with some strains of *P. freudenreichii* in an in vitro batch culture study that used a substrate of either 100% ground corn or mixed-forage (25% barley silage, 25% alfalfa hay, and 50% grass hay). Alazzeh et al. (2012) did not observe consistent CH_4_ reduction nor lower CH_4_ production consistently accompanied by increase in molar proportion of propionate for all evaluated *Propionibacterium* strains, and the results were attributed to differences in strain of DFM used. In addition, differences in the substrates used may also explain inconsistent responses as previous authors (Alazzeh et al., 2012; Philippeau et al., 2017) have observed reduced CH_4_ production when low concentrate or lower starch diets were fed. Alazzeh et al. (2012) observed reduced CH_4_ production either with substrate of ground corn or forage when some strains of *P. freudenreichii* were supplemented; the authors however observed reduced CH_4_ production only with the forage substrate for other strains of *P. freudenreichii*. Similarly, Philippeau et al. (2017) reported reduced CH_4_ production per kg milk produced when a low-starch diet (2.2% starch) was fed to lactating dairy cows, but not with a high-starch diet (38.2% starch). This selective effect of DFM on CH_4_ concentration as observed in these studies might be due to a higher likelihood of higher CH_4_ concentration with diets rich in NDF, given such diet results in higher molar proportion of acetate, which is accompanied by higher production of CO_2_, a precursor for CH_4_ production by methanogens.

An increase in the molar proportion of propionate is often at the cost of decreasing the proportion of either acetate or butyrate. Both PRO-A and PRO-B lowered the molar proportion of butyrate, but this did not translate towards a greater molar proportion of propionate. This lack of difference in proportion of propionate might be due to insufficient inoculation dose, lack of viability, or timing of rumen sampling relative to feeding (Stein et al., 2006; Vyas et al., 2014b).

## CONCLUSIONS

This study explored the impact of DFM on rumen fermentation parameters and nutrient degradability in dairy cows using different methodologies with rumen fluid from lactating Holstein dairy cows. Overall, DFM increased in vitro DM and NDF digestibility when using rumen fluid from cows not previously exposed to DFM but not from DFM-consuming cows. This highlights the complexity of DFM-substrate interactions in the rumen and the need for further research to understand how DFM supplementation influences rumen fermentation in dairy cows. Additional studies are warranted to understand how DFM supplementation is impacted under different production settings, including dietary profile and physiological state of the cow herd.

## Supplementary Data

Supplementary data are available at *Translational Animal Science* online.

txae162_suppl_Supplementary_Materials

## Abbreviations:

CH_4_: methane
DFM: direct-fed microbial
DM: dry matter
GP: gas pressure
GV: gas volume
IVDMD: in vitro dry matter digestibility
IVNDFD: in vitro neutral detergent fiber digestibility
NDF: neutral detergent fiber
TMR: total mixed ration
VFA: volatile fatty acid

## References

[CIT0001] Alazzeh, A., H. Sultana, K. Beauchemin, Y. Wang, H. Holo, O. Harstad, and T. McAllister. 2012. Using strains of Propionibacteria to mitigate methane emissions in vitro. Acta Agri. Scand. 62:263–272. doi: https://doi.org/10.1080/09064702.2013.773056

[CIT0004] Boyd, J., J. West, and J. Bernard. 2011. Effects of the addition of direct-fed microbials and glycerol to the diet of lactating dairy cows on milk yield and apparent efficiency of yield. J. Dairy Sci. 94:4616–4622. doi: https://doi.org/10.3168/jds.2010-3984 21854934

[CIT0005] Cappellozza, B. I., J. N. Joergensen, G. Copani, K. A. Bryan, P. Fantinati, J. -C. Bodin, M. Malek Khahi, C. NinoDeGuzman, K. G. Arriola, L. O. Lima, et al. 2023a. Evaluation of a *Bacillus*-based direct-fed microbial probiotic on *in vitro* rumen gas production and nutrient digestibility of different feedstuffs and total mixed rations. Transl. Anim. Sci. 7:txad044. doi: https://doi.org/10.1093/tas/txad044 37216187 PMC10199785

[CIT0006] Cappellozza, B. I., A. Segura, N. Milora, C. Galschioet, M. Schjelde, and G. Copani. 2023b. Stability of *Bacillus* and *Enterococcus faecium* 669 probiotic strains when added to different feed matrices used in dairy production. Animals 13:2350. doi: https://doi.org/10.3390/ani13142350 37508127 PMC10375954

[CIT0007] Chen, J., O. M. Harstad, T. McAllister, P. Dörsch, and H. Holo. 2020. Propionic acid bacteria enhance ruminal feed degradation and reduce methane production in vitro. Acta Agric. Scand. A Anim. Sci. 69:169–175. doi: https://doi.org/10.1080/09064702.2020.1737215

[CIT0008] Dhakal, R., G. Copani, B. I. Cappellozza, N. Milora, and H. H. Hansen. 2023. The effect of direct-fed microbials on in vitro rumen fermentation of grass or maize silage. Fermentation 9:347. doi: https://doi.org/10.3390/fermentation9040347

[CIT0009] Dickey, C. E. 2016. Effect of bovamine on ruminal, post-ruminal, and total tract digestibilities in dairy cows and on animal performance [MSc]. Columbus, OH: The Ohio State University.

[CIT0010] Ferraretto, L. F., A. C. Fonseca, C. J. Sniffen, A. Formigoni, and R. D. Shaver. 2015. Effect of corn silage hybrids differing in starch and neutral detergent fiber digestibility on lactation performance and total-tract nutrient digestibility by dairy cows. J. Dairy Sci. 98:395–405. doi: https://doi.org/10.3168/jds.2014-8232 25465561

[CIT0011] Ferraretto, L. F., and R. Shaver. 2015. Effect of direct-fed microbial supplementation on lactation performance and total-tract starch digestibility by midlactation dairy cows. Prof. Anim. Sci. 31:63–67. doi: https://doi.org/10.15232/pas.2014-01369

[CIT0012] Goering, H. K., and P. J. Van Soest. 1970. Forage fiber analyses (apparatus, reagents, procedures, and some applications). ARS/USDA Handbook No. 379, Superintendent of Documents, US Government Printing Office, Washington, D.C. 20402.

[CIT0013] Hall, M. B. 2015. Determination of dietary starch in animal feeds and pet food by an enzymatic-colorimetric method: collaborative study. J. AOAC Int. 98:397–409. doi: https://doi.org/10.5740/jaoacint.15-012 25905746

[CIT0014] Huntington, G. 1990. Energy metabolism in the digestive tract and liver of cattle: influence of physiological state and nutrition. Reprod. Nutr. Dev. 30:35–47. doi: https://doi.org/10.1051/rnd:19900103 2184823

[CIT0016] Jeyanathan, J., C. Martin, and D. Morgavi. 2014. The use of direct-fed microbials for mitigation of ruminant methane emissions: a review. Animal 8:250–261. doi: https://doi.org/10.1017/s1751731113002085 24274095

[CIT0017] Jeyanathan, J., C. Martin, and D. P. Morgavi. 2016. Screening of bacterial direct-fed microbials for their antimethanogenic potential in vitro and assessment of their effect on ruminal fermentation and microbial profiles in sheep. J. Anim. Sci. 94:739–750. doi: https://doi.org/10.2527/jas.2015-9682 27065144

[CIT0019] Krehbiel, C. R., S. R. Rust, G. Zhang, and S. E. Gilliland. 2003. Bacterial direct-fed microbials in ruminant diets: performance response and mode of action. J. Anim. Sci. 81:E120–E132. doi: https://doi.org/10.2527/2003.8114_suppl_2E120x

[CIT0020] Lawrence, M., S. Polukis, A. Barnard, M. Miller, L. Kung, Jr, and T. Gressley. 2021. Evaluating the effects of *Lactobacillus animalis* and *Propionibacterium freudenreichii* on performance and rumen and fecal measures in lactating dairy cows. J. Dairy Sci. 104:4119–4133. doi: https://doi.org/10.3168/jds.2020-19291 33612206

[CIT0021] Maderal, A., I. Fernandez-Marenchino, W. Cuervo, F. Tarnonsky, F. Podversich, J. J. V. Martinez, M. R. Moreno, T. M. Schulmeister, O. Queiroz, B. Cappellozza, et al. 2022. Effect of increasing supplementation of bacillus spp. on in vitro ruminal fermentation and nutrient digestibility. J. Anim. Sci. 100:359. doi: https://doi.org/10.1093/jas/skac247.655

[CIT0022] Mauricio, R. M., F. L. Mould, M. S. Dhanoa, E. Owen, K. S. Channa, and M. K. Theodorou. 1999. A semi-automated in vitro gas production technique for ruminant feedstuff evaluation. Anim. Feed Sci. Technol. 79:321–330. doi: https://doi.org/10.1016/s0377-8401(99)00033-4

[CIT0023] McAllister, T., K. Beauchemin, A. Alazzeh, J. Baah, R. Teather, and K. Stanford. 2011. The use of direct fed microbials to mitigate pathogens and enhance production in cattle. Can. J. Anim. Sci. 91:193–211. doi: https://doi.org/10.4141/cjas10047

[CIT0024] Meale, S. J., S. Ding, M. L. He, M. E. R. Dugan, G. O. J. Ribeiro, A. Y. Alazzeh, H. Holo, O. M. Harstad, T. A. McAllister, and A. V. Chaves. 2014. Effect of Propionibacterium freudenreichii on ruminal fermentation patterns, methane production and lipid biohydrogenation of beef finishing diets containing flaxseed oil in a rumen simulation technique. Can. J. Anim. Sci. 94:685–695. doi: https://doi.org/10.4141/cjas-2014-051

[CIT0048] McDougall EI. Studies on ruminant saliva. 1. The composition and output of sheep’s saliva. Biochem J. 1948;43(1):99-109. PMID: 16748377; PMCID: PMC1274641. PMC1274641 16748377

[CIT0025] Monteiro, H. F., A. L. J. Lelis, V. L. N. Brandao, A. Faccenda, A. S. Avila, J. Arce-Cordero, L. G. Silva, X. Dai, R. Restelatto, P. Carvalho, et al. 2019. In vitro evaluation of *Lactobacillus plantarum* as direct-fed microbials in high-producing dairy cows diets. Transl Anim Sci 4:214–228. doi: https://doi.org/10.1093/tas/txz187 32704981 PMC6994042

[CIT0026] Moss, A. R., J.-P. Jouany, and J. Newbold. 2000. Methane production by ruminants: its contribution to global warming. In: Proceedings of the Annales de zootechnie. EDP Sciences. p. 231–253.

[CIT0027] NRC. 2001. Nutrient requirements of dairy cattle. (7th rev. ed.). Washington, DC: Natl. Acad. Press.

[CIT0028] Oyebade, A. O., S. Lee, H. Sultana, K. Arriola, E. Duvalsaint, C. Nino De Guzman, I. Fernandez Marenchino, L. Marroquin Pacheco, F. Amaro, L. Ghedin Ghizzi, et al. 2023a. Effects of direct-fed microbial supplementation on performance and immune parameters of lactating dairy cows. J. Dairy Sci. 106:8611–8626. doi: https://doi.org/10.3168/jds.2022-22898 37641244

[CIT0029] Oyebade, A. O., G. A. Taiwo, M. Idowu, T. Sidney, D. Vyas, and I. M. Ogunade. 2023b. A multi-species direct-fed microbial supplement alters the milk lipidome of dairy cows. JDS Commun. 4:25–30. doi: https://doi.org/10.3168/jdsc.2022-0244 36713121 PMC9873687

[CIT0030] Pan, L., K. Harper, O. Queiroz, G. Copani, and B. I. Cappellozza. 2022. Effects of a *Bacillus*-based direct-fed microbial on *in vitro* nutrient digestibility of forage and high-starch concentrate substrates. Transl. Anim. Sci. 6:1–9. doi: https://doi.org/10.1093/tas/txac067 PMC9186312 35702175

[CIT0031] Pech-Cervantes, A. A., I. M. Ogunade, Y. Jiang, M. Irfan, K. G. Arriola, F. X. Amaro, C. F. Gonzalez, N. DiLorenzo, J. J. Bromfield, D. Vyas, et al. 2019. An expansin-like protein expands forage cell walls and synergistically increases hydrolysis, digestibility and fermentation of livestock feeds by fibrolytic enzymes. PLoS One 14:e0224381. doi: https://doi.org/10.1371/journal.pone.0224381 31689330 PMC6830940

[CIT0032] Philippeau, C., A. Lettat, C. Martin, M. Silberberg, D. Morgavi, A. Ferlay, C. Berger, and P. Nozière. 2017. Effects of bacterial direct-fed microbials on ruminal characteristics, methane emission, and milk fatty acid composition in cows fed high-or low-starch diets. J. Dairy Sci. 100:2637–2650. doi: https://doi.org/10.3168/jds.2016-11663 28161181

[CIT0033] Qiao, G. H., A. S. Shan, N. Ma, Q. Q. Ma, and Z. W. Sun. 2010. Effect of supplemental *Bacillus* cultures on rumen fermentation and milk yield in Chinese Holstein cows. J. Anim. Physiol. Anim. Nutr. 94:429–436. doi: https://doi.org/10.1111/j.1439-0396.2009.00926.x 19663976

[CIT0034] Raeth-Knight, M., J. Linn, and H. Jung. 2007. Effect of direct-fed microbials on performance, diet digestibility, and rumen characteristics of Holstein dairy cows. J. Dairy Sci. 90:1802–1809. doi: https://doi.org/10.3168/jds.2006-643 17369221

[CIT0035] Romero, J. J., M. A. Zarate, and A. T. Adesogan. 2015. Effect of the dose of exogenous fibrolytic enzyme preparations on preingestive fiber hydrolysis, ruminal fermentation, and in vitro digestibility of bermudagrass haylage. J. Dairy Sci. 98:406–417. doi: https://doi.org/10.3168/jds.2014-8285 25468699

[CIT0036] Saylor, B. A., T. Fernandes, H. Sultana, A. Gallo, and L. F. Ferraretto. 2020. Influence of microbial inoculation and length of storage on fermentation profile, N fractions, and ruminal in situ starch disappearance of whole-plant corn silage. Anim. Feed Sci. Technol. 267:114557. doi: https://doi.org/10.1016/j.anifeedsci.2020.114557

[CIT0037] Schallmey, M., A. Singh, and O. P. Ward. 2004. Developments in the use of *Bacillus* species for industrial production. Can. J. Microbiol. 50:1–17. doi: https://doi.org/10.1139/w03-076 15052317

[CIT0038] Seo, J. -K., S. -W. Kim, M. -H. Kim, S. D. Upadhaya, D. -K. Kam, and J. -K. Ha. 2010. Direct-fed microbials for ruminant animals. Asian-Aust. J. Anim. Sci. 23:1657–1667. doi: https://doi.org/10.5713/ajas.2010.r.08

[CIT0039] Silva, T. H., B. R. Amâncio, E. Magnani, G. W. Meurer, H. G. Reolon, T. G. Timm, B. I. Cappellozza, R. H. Branco, and E. M. Paula. 2024. Evaluation of direct-fed microbials on in vitro ruminal fermentation, gas production kinetic, and greenhouse gas emissions in different ruminants’ diet. Front. Anim. Sci. 5:1320075. doi: https://doi.org/10.3389/fanim.2024.1320075

[CIT0040] Stein, D. R., D. T. Allen, E. B. Perry, J. C. Bruner, K. W. Gates, T. G. Rehberger, K. Mertz, D. Jones, and L. J. Spicer. 2006. Effects of feeding propionibacteria to dairy cows on milk yield, milk components, and reproduction. J. Dairy Sci. 89:111–125. doi: https://doi.org/10.3168/jds.S0022-0302(06)72074-4 16357273

[CIT0041] Sun, P., J. Wang, and L. Deng. 2013. Effects of *Bacillus subtilis* natto on milk production, rumen fermentation and ruminal microbiome of dairy cows. Animal 7:216–222. doi: https://doi.org/10.1017/s1751731112001188 23031615

[CIT0042] Ungerfeld, E. M. 2020. Metabolic hydrogen flows in rumen fermentation: principles and possibilities of interventions. Front. Microbiol. 11:589. doi: https://doi.org/10.3389/fmicb.2020.00589 32351469 PMC7174568

[CIT0043] Van Soest, P., J. Robertson, and B. Lewis. 1991. Methods for dietary fiber, neutral detergent fiber, and nonstarch polysaccharides in relation to animal nutrition. J. Dairy Sci. 74:3583–3597. doi: https://doi.org/10.3168/jds.s0022-0302(91)78551-2 1660498

[CIT0044] Vyas, D., A. Alazzeh, S. M. McGinn, T. A. McAllister, O. M. Harstad, H. Holo, and K. A. Beauchemin. 2015. Enteric methane emissions in response to ruminal inoculation of Propionibacterium strains in beef cattle fed a mixed diet. Anim. Prod. Sci. 56:1035–1040. doi: https://doi.org/10.1071/an14801

[CIT0045] Vyas, D., E. J. McGeough, S. M. McGinn, T. A. McAllister, and K. A. Beauchemin. 2014a. Effect of *Propionibacterium* spp. on ruminal fermentation, nutrient digestibility, and methane emissions in beef heifers fed a high-forage diet. J. Anim. Sci. 92:2192–2201. doi: https://doi.org/10.2527/jas.2013-7492 24663192

[CIT0046] Vyas, D., E. J. McGeough, R. Mohammed, S. M. McGinn, T. A. McAllister, and K. A. Beauchemin. 2014b. Effects of *Propionibacterium* strains on ruminal fermentation, nutrient digestibility and methane emissions in beef cattle fed a corn grain finishing diet. Animal 8:1807–1815. doi: https://doi.org/10.1017/S1751731114001657 25322788

[CIT0047] West, J., and J. Bernard. 2011. Effects of addition of bacterial inoculants to the diets of lactating dairy cows on feed intake, milk yield, and milk composition. Prof. Anim. Sci. 27:122–126. doi: https://doi.org/10.15232/s1080-7446(15)30458-7

