# Supplementary material for: Effects of direct-fed microbials supplementation on in vitro and ex vivo ruminal fermentation and nutrient degradability in lactating Holstein dairy cows
Source: Transl Anim Sci. 2024 Dec 14;8:txae162. doi: 10.1093/tas/txae162 (PMC11657564; doi:10.1093/tas/txae162)
Supplement: txae162_suppl_Supplementary_Materials [file txae162_suppl_supplementary_materials.docx]

**Materials and Methods (Feeding study)**

**Measurements**

*Body weight and milk parameters.* Cows were automatically weighed twice daily after each milking, on a walk-in electronic scale (Afi-Weigh, S.A.E. Afikim, Israel) located on the exit lane of the milking parlor. Daily BW values were collected for all cows from d 15 to 28 of each period. Daily milk yield data were collected for cows from d 15 to 28 and milk samples were collected from morning and evening milking of d 15, 17, 22, and 24 of each period. Milk samples were sent to Southeast Milk Dairy Laboratory (Bellevue, FL) for determination of milk fat, true protein, lactose, and milk urea nitrogen (MUN) concentrations using a Fourier Transform Spectrometer model FTS 500 (Bentley Instruments Inc., Chaska, MN).

*Intake and Digestibility.* The amount of feed offered, and orts were measured daily and recorded for individual cows. Approximately 200 g of TMR offered and left-over were collected from each cow on d 22, 23, and 24 of each period. Fecal grab samples were collected every 3-h interval starting at 0500 h, 0600 h and 0700 h on d 22, 23, and 24, respectively. Both TMR, refusals, and fecal samples were dried in a forced-air oven at 55°C for 48 h. Dried samples were ground in a Willey mill (A.H. Thomas Scientific, Philadelphia, PA) to pass through a 1.0-mm sieve and composited per cow per period for digestibility analysis. Indigestible NDF (**iNDF**) was measured on all samples and used as internal marker to estimate digestibility coefficients. The iNDF evaluation was carried out by adding 0.7 g of each sample to F-57 Ankom bags, which were incubated *in situ* for 288 h ([Ahvenjärvi et al., 2000](#_ENREF_1)). Samples were analyzed for DM (105°C for 12 h), CP, EE, and NDF digestibility. Nitrogen content was measured by rapid combustion using a Macro Elemental N analyzer ([AOAC, 2000; Vario MAX CN, #25.00-5003; Elementar, Hanau, Germany](#_ENREF_3)). The N values were multiplied by 6.25 to calculate the CP composition for each sample. Analysis of NDF was done with an Ankom-200 Fiber Analyzer (Ankom Technologies, Macedon, NY) using heat-stable α-amylase and sodium sulfite ([aNDF; method 2002.04, AOAC, 2012](#_ENREF_4)). Ether extract content was determined by solvent extraction, using an Ankom XT15 extractor (Ankom Technologies, Macedon, NY).

*Urine and plasma parameters.* Urine samples were collected twice daily on d 23 and 24 of each period. Samples were filtered through two layers of cheese cloth and 20 mL of filtered urine was mixed with 80 mL 0.036 N sulfuric acid and stored at -20°C for later analysis of allantoin, uric acid, creatinine, and urine N. Urine samples were sent to the Forage Evaluation Support Laboratory (Gainesville, FL, USA) for urinary N estimation. Concentration of allantoin was determined colorimetrically using the procedure described by [Chen and Gomes (1992)](#_ENREF_8). Uric acid was determined using the Infinity Uric Acid Liquid Stable Reagent (Thermo Fisher Scientific Inc., Middletown, VA, USA), whereas creatinine was determined using a colorimetric kit (Arbor Assays; Ann Arbor, MI, USA). The average daily urine output was estimated using creatinine concentration as a marker and assuming daily creatinine excretion of 29 mg/kg BW ([Cobianchi et al., 2012](#_ENREF_9)). Total purine derivative (**PD**) concentration was calculated as the sum of urinary concentration of allantoin and uric acid, while the absorbed purines (**AP**, mmol/d) was calculated as a function of excreted PD, using the equation: PD = 0.85 × AP + 0.512 × BW^0.75^, as described by [Cobianchi et al. (2012)](#_ENREF_9).

Blood samples (20 mL) were collected from the coccygeal vein at 0800 h on d 25 of each period, using vacutainers containing sodium heparin (Becton Dickinson, Franklin Lakes, NJ, USA). Blood samples were kept on ice immediately after collection, and within 20 min, samples were centrifuged at 2,500 × *g* for 20 min at 4°C to harvest plasma fraction. Plasma was transferred into snap cap microcentrifuge tubes (Eppendorf AG, Hamburg, Germany) and stored at -20°C for later analysis of glucose, non-esterified fatty acids (**NEFA**), blood urea-N (**BUN**), and β-hydroxybutyrate (**BHB**). Plasma glucose concentration was determined using an enzymatic assay (Teco Diagnostics, Anaheim, CA, USA). Plasma NEFA and BHB concentrations were determined using a NEFA assay reagent kit (NEFA-HR, Fujifilm Wako Diagnostics U.S.A. Corp., Mountain View, CA, USA) and the Autokit 3-HB Assay kit (Fujifilm Wako Diagnostics U.S.A. Corp., Montain View), respectively. Blood urea-N concentration was estimated using a colorimetric detection kit (Arbor Assays, Ann Arbor).

*Rumen sampling.* Rumen fluid was collected from each cow on d 15 of each period at 0, 3, 6, 9, 12, and 24 h after feeding. Approximately 600 mL of rumen fluid was collected through the cannula. A subsample (40 mL) of the rumen fluid was strained through 4 layers of cheesecloth and after pH measurement, sample was stored at -20°C for ammonia nitrogen (**NH_3_-N**) analysis at each time point.
